# Factors Determining the Burden of a Caregiver Providing Care to a Post-Stroke Patient

**DOI:** 10.3390/jcm14093008

**Published:** 2025-04-26

**Authors:** Bogusława Ryś, Ewelina Bąk

**Affiliations:** Faculty of Health Sciences, University of Bielsko-Biala, ul.Willowa 2, 43-309 Bielsko-Biala, Poland; brys@ubb.edu.pl

**Keywords:** caregiver burden, determinants, stroke rehabilitation, quality of life

## Abstract

**Background**: Physical, emotional, psychological, and social factors influence the high level of burden of a caregiver providing care for a patient at home after a stroke. The purpose of this study was to identify and evaluate factors influencing the high level of burden on the caregiver providing care for a post-stroke patient, including factors on the part of the patient and caregiver. **Methods**: This cross-sectional study was conducted at the Neurological Rehabilitation Department of the Hospital Beskid Treatment and Rehabilitation Complex in Jaworze, Poland, and the Neurological Rehabilitation Department of the Railway Hospital in Wilkowice-Bystra. The study participants comprised post-stroke patients and their family caregivers (during visits to hospital), 110 pairs. The measures for caregivers were such as the following: Beck Depression Inventory, the Polish adaptation of the Perceived Stress Scale, the Polish adaptation of the Mini-COPE questionnaire to measure stress coping strategies, and the WHO Quality of Life Brief Version. The measures for patients were such as the following: the modified Rankin Scale and Abbreviated Mental Test Score to assess functional capacity for simple Activities of Daily Living (ADL). All statistical calculations were performed using the R statistical package version 4.4.2. **Results:** A high caregiver burden was found in 30 people (27.3%). Logistic regression analysis proved that low quality of life, stress, caregiver-triggered strategies (discharge and cessation of activities), caregiver frustration, psychological burden, financial situation, longer time spent on patient care, functional status (ADL) on the part of the patient, judgment of significant degree of disability judgment, and age of the patient are determinants affecting high caregiver burden levels. **Conclusions**: Almost 1/3 of caregivers experienced a high burden when taking care of a person after stroke. Analyzing the Gini index, from the model’s point of view, quality of life is the most important characteristics, and caregiver frustration is the least important, which influences the high level of caregiver burden.

## 1. Introduction

The Global Burden of Diseases, Injuries, and Risk Factors Study (GBD) 2017 showed that stroke was the third-leading cause of death and disability combined (as measured by disability-adjusted life-years [DALYs]) and the second-leading cause of death in the world in 2017 [1,2]. Since a majority of the survivors return home, their family caregivers, mainly their partners, also experience the social repercussions of stroke, such as whether they receive expressions of sympathy, whether friendship bonds are strengthened, whether they lose friends, and whether their social life is maintained as before. Expected to provide complex care at home in addition to having new responsibilities (increased home tasks and duties, management of relationships with professionals, etc.), these dependency workers [3] pose a risk for the healthcare system while their exhaustion increases and their cultural, social, and leisure activities decrease [4,5]. This workload, in addition to their continual adaptation to the limitations of the patient, may isolate them socially. Thus, when they themselves are affected by the repercussions of the stroke as the caregiver, their capacity to remain healthy becomes a challenge for public health and health policies [6,7]. A family caregiver is a family member, such as a spouse or child who provides care to a person with a chronic illness who needs assistance with daily living tasks and taking medicine [8]. However, family caregivers often must assume their new role suddenly, resulting in changes in the family functioning pattern [9]. During this period, a family caregiver must face new problems with a possible sense of inadequacy due to a lack of the knowledge and skills necessary to carry out the role of care and assistance, such as managing medications, preparing food, and supporting the patient [10]. The intense stress caused by the hard work of care and assistance over a long period of time has been defined as a “family burden” [11]. This study fits into one of the key targets specified in the Action Plan for Stroke in Europe 2018–2030. Four overarching targets for 2030 were identified: to reduce the absolute number of strokes in Europe by 10%, to treat 90% or more of all patients with stroke in Europe in a dedicated stroke unit as the first level of care, to have national plans for stroke encompassing the entire chain of care, and to fully implement national strategies for multisector public health interventions [12]. When we consider vascular diseases, which are a public health and social policy priority in Europe, the importance of cardiovascular diseases should also be emphasized [13]. Lee et al. [14] presented a study considering high burdens such as disability, stress, and quality of life.

The purpose of this study was to identify and evaluate factors influencing the high level of burden on the caregiver providing care for a post-stroke patient, including factors on the part of the patient and caregiver.

## 2. Materials and Methods

### 2.1. Organization of the Study

**Figure 1:**
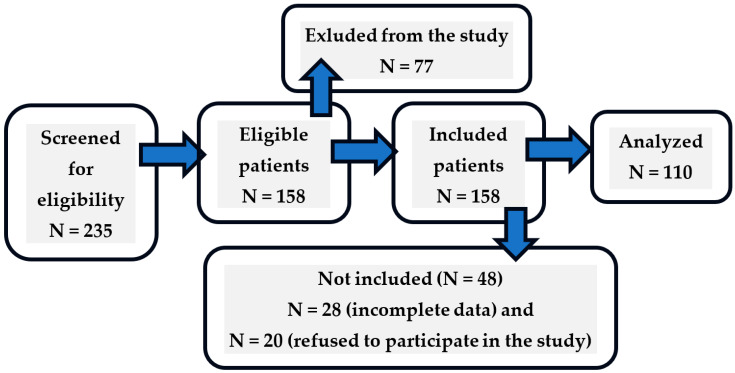
Flowchart of caregivers of stroke patient.

This cross-sectional study was conducted at the Neurological Rehabilitation Department of the Hospital Beskid Treatment and Rehabilitation Complex in Jaworze, Poland, and the Neurological Rehabilitation Department of the Railway Hospital in Wilkowice-Bystra, Poland, from 10 April 2024 to 20 February 2025. Researcher B.R. provided questionnaires to patients, as well as their caregivers, after obtaining their informed, voluntary consent. The study participants comprised post-stroke patients and their family caregivers (during visits to hospital), 110 pairs. The wards in which the study was conducted had 37 beds in Jaworze and 21 beds in Wilkowice. Patients admitted for late neurological rehabilitation during the study period from 10 April 2024 to 20 February 2025 were 155 in number in Jaworze and 80 in Wilkowice. A total of 235 respondents. Figure 1 describes that in detail. Of all 235 patients hospitalized in this period at the Neurological Rehabilitation Department, patients who met the eligibility criteria for the study qualified (living in the same house as the patient for at least six month after ischemic stroke). The recruitment process is presented Figure 1.

### 2.2. Inclusion and Exclusion Criteria

The following inclusion criteria were used for patients: the researcher recruited patients with ischemic stroke at least six months after stroke and living in the same house, age > 35, with patient consent to participate in the study. Exclusion criteria: age < 35, less than 6 months since the diagnosis of ischemic stroke, and lack of consent to participate in the study.

The following inclusion criteria were used for caregivers: living in the same house with patient for at least six months after stroke, age > 20, and consent to participate in the study. Exclusion criteria: age < 20, lack of consent to participate in the study, and not living in the same house.

Caregiver measures:

- The Caregiver Burden Scale (CBS) scale using a 22-item scale that was developed in Sweden [15] and is divided into five components as a result of the previous factor analysis: general strain, isolation, disappointment, emotional involvement, and environment. The items are scored from 1 to 4 (never, rarely, sometimes, and always, respectively) and cover questions about caregivers’ health, feelings of psychological well-being, relationships, social networks, physical workload, and environmental aspects. The total burden index consists of the average of all 22 items, with higher scores indicating a greater level of burden. In accordance with the recommendations of the scale’s authors, the following load categories were adopted: low (1.00–1.99 points), medium (2.00–2.99 points), and high (3.00–4.00 points). The results proved to have good design accuracy. There was adequate internal consistency for all subscales (α-Cronbach = 0.70–0.87), and the research by Jaracz et.al.: α-Cronbach = 0.92 [16]. Own research: α-Cronbach = 0.96;
- For information on the caregiver’s perception of QoL, the World Health Organization Quality of Life (WHOQOL-BREF) questionnaire was used. This instrument consists of four domains and aims to verify psychological well-being, physical capacity, social relationships, and the environment where the individual is inserted, containing a total of 26 questions. It also presents two more general questions about QoL. Higher scores indicate a better QoL. Responses were rated on a 5-point Likert scale [17]. Own research: α-Cronbach = 0.94;
- Beck Depression Inventory (BDI) The BDI is a 21-point screening questionnaire that is used to assess the severity of mood disorder (depression) symptoms. The scale consists of 21 questions that score from 0 to 3 points. The results that can be obtained in the BDI range from 0 to 63. The BDI is a questionnaire that has been standardized and validated to Polish conditions, and it is repeatedly used in studies to assess mood disorders [18]. Own research: α-Cronbach = 0.92;
- To assess the intensity of perceived stress among caregivers, we used the Polish adaptation of the Perceived Stress Scale (PPS-10) questionnaire [19], the original version of which was developed by Cohen et al. [20] and which contains 10 questions on various subjective feelings related to personal problems and events, behaviors, and ways of coping. In order to compare the results of the surveys, the overall PSS-10 scores were normalized (based on [19]), which took into account gender, age, education level, and occupation. The surveys were conducted in various locations in Central Poland. The normalization was carried out on a sample of healthy people numbering 1830. The respondents gave their answers by writing the appropriate number (0—never, 1—almost never, 2—sometimes, 3—quite often, 4—very often). The overall score of the scale is the sum of all the scores, the theoretical distribution of which is from 0 to 40. The higher the score, the greater the severity of the perceived stress. The overall index, after conversion to standardized units, is subject to interpretation according to the properties that characterize the so-called stena scale i.e., values on a scale of 1–10. Scores within 1–4 stena are treated as low scores, while scores within 7–10 stena are treated as high scores. Scores within 5–6 stena are treated as average, α-Cronbach’s > 70 [21]. Own research: α-Cronbach = 0.70;
- The Polish adaptation of the Mini-COPE questionnaire—a shortened version of the Coping Inventory-Mini-COPE [19], the original version of which was developed by C. S. Carver [22]—was used to measure coping strategies. The questionnaire is an abbreviated version of the Multidimensional Coping Inventory-COPE and consists of 28 statements comprising 14 coping strategies. Each scale includes 2 statements and 5 groups of strategies, to which the respondent responds on a scale from 0 (I almost never do this) to 3 (I almost always do this). The higher the score, the more often the respondent uses a particular strategy. Own research: α-Cronbach = 0.75;

Patient measures:

- The modified Rankin Scale was used to measure the disability of post-stroke individuals. This scale has six categories ranging from 0 (no symptoms) to 5 (severe disability) [23]. It is a systematic method of assessing the patient’s ability, identifying skills or deficiencies in self-care. The modified Rankin Scale is a simple instrument with good acceptability for measuring the level of functional recovery of post-stroke individuals and used as a scale for activities of daily living (ADL). The participants’ sociodemographic data and the patients’ clinical information were gathered from the medical records and with the help of a semi-structured questionnaire.
- The Abbreviated Mental Test Score (AMTS), is a tool designed for the assessment of cognitive functions.;It consists of 10 short tasks—questions and commands addressed to the subject. For a correct answer or execution of a command, the examinee receives 1 point. The maximum number of points possible is 10, the minimum—0. On the basis of the obtained score, the efficiency of the cognitive functions of the person examined is assessed, classifying them into one of three levels of mental efficiency: a score of 7–10 points means a normal state, a score of 4–6 points—moderate impairment, and a score of 0–3 points—severe impairment of cognitive functions. The Polish adaptation of the questionnaire was used [24], the original version of which was developed by [25].

### 2.3. Statistical Methods

Continuous variables were expressed by mean, standard deviation and quartiles, while qualitative variables were expressed by counts and percentages. Kendall’s tau coefficient and Spearman’s R were used to test the relationship between quantitative variables. Logistic regression models were analyzed, where the explanatory variable took two values: “1”—caregivers with high levels of burden, ‘0’—caregivers with low and medium levels of burden. The likelihood ratio test, Hosmer–Lemeshow test, and Akaike information criterion were used to test the statistical significance of all parameters (explanatory variables). A measure proposed by McFadden (McFadden’s pseudo R-square) was used to assess model fit. Parameters in the models were interpreted using the odds ratio. The validity of a given model parameter was determined using the Gini index. The ROC curve was used to determine the quality of the model, where the AUC was marked as a measure of the goodness and relevance of the model. An important part of the analysis was the determination of three measures based on the relevance table, i.e.,: effectiveness (accuracy), sensitivity, and specificity. All statistical calculations were performed using the R statistical package version 4.4.2

## 3. Results

### 3.1. Participant Characteristics Patients/Caregivers

A total of 110 pairs (110 post-stroke patients, 110 family caregivers) participated in late neurological rehabilitation in the neurological rehabilitation unit, after ischemic stroke. Patients’ comorbidities were as follows: hypertension 106 (96.4%), atherosclerosis 64 (58.2%), hypercholesterolemia 41 (37.3%), type 2 diabetes mellitus 36 (32.7%), atrial fibrillation 27 (24.5%), and nicotinism 26 (23.6%). Knowledge of stroke: affected hemibody: right 29 (26.4%), left 49 (36%), speech symptoms (dysartria) 40 (36.4%), difficulty performing daily activities 71 (64.5%), difficulty swallowing 18 (16.4%). Caregiver comorbidities: hypertension 57 (51.8%), myasthenia gravis 13 (11.8%). Diabetes 19 (17.3%), neurosis 13 (11.8%). Caregiver fatigue was most often described by caregivers as ‘I often feel tired during the day’ 74 (67.3), ‘I constantly feel tired’ 13 (11.8%), ‘I feel exhausted’ 8 (7.3). To the question asked about mental state, caregivers answered as follows: ‘I often feel anxious’ 64 (58.2%), ‘I often feel sad and depressed’ 21 (19.1%), ‘I am often upset’ 15 (13.6%), ‘I feel frustration and anger’ 8 (7.35), ‘I feel mentally exhausted’ 10 (9.3%). Detailed data are shown in Table 1.

### 3.2. Caregiver Burden According to CBS Scale

The average total burden score according to the CB Scale was 2.39, indicating a medium level of burden. The score was highest in the “overall burden” subscale, and the lowest scores were in the “emotional involvement” and “environment” subscales According to the accepted criterion, 30 caregivers (27.3%) felt a high level of burden. Detailed data are shown in Table 2.

### 3.3. Determinants of Caregiver Burden

CBS scale domains correlate significantly positively (*p* < 0.001, *p* < 0.01, *p* < 0.05) with the PSS-10, Beck, and Rankin scales and thus the greater the severity of stress and depression and greater functional disability, the greater the caregiver burden. (Table 3). In contrast, the domains of the CBS scale correlate significantly negatively (*p* < 0.001, *p* < 0.01, *p* < 0.05) with the WHQOL-BREF, ADL, and AMTS scale. This means that the lower the efficiency of activities of daily living, the lower the efficiency of cognitive functions, and the lower the quality of life of caregivers and the higher the burden on the caregiver. The domains of the CBS scale correlate significantly positively with the Mini-COPE scale at the level of (*p* < 0.001) in the domains of denial, cessation of actions, and blaming oneself, which means that the more often the domains are touched on in the Mini-COPE, the higher the caregiver burden (Table 3).

### 3.4. Multivariable Regression Analysis with Significant Factors Was Performed on High-Burden Caregiver

The following were identified as independent high caregiver burden predictors in stroke patients: PSS-10 odds ratio (OR) = 1.198; 95% confidence interval (CI) 1.096–1.334; (<0.0001) It can be concluded that stress increases the chance of a high burden by 19.8%. ADL odds ratio (OR) = 0.646; 95% confidence interval (CI) 0.458–0.863; (<0.006); with fully preserved activities of daily living, the chance of high burden on the caregiver is 35.4% lower, WHOQoL odds ratio (OR) = 0.928; 95% confidence interval (CI) 0.889–0.963; (<0.0001); the higher the level of quality of life of the caregiver, the chance of high burden is 7.2% lower. Analyzing the Gini coefficient, it can be concluded that from the point of view of the model, the most relevant characteristic is the caregiver’s quality of life. In the study of stress level, the influence of individual domains of Mini-Cope (Model I) and individual domains of WHOQoL quality of life (Model II) was examined. Model I shows the influence of 2 domains out of 14 on the caregiver’s high stress level, i.e., discharge odds ratio (OR) = 2.335; 95% confidence interval (CI) 1.134–5.115; (<0.0254); and cessation of activities odds ratio (OR) = 3.699; 95% confidence interval (CI) 1.750–8.785; (<0.0013). These characteristics contribute to the likelihood of a high-stress caregiver caring for a post-stroke patient. Disclosure of a caregiver’s negative emotions increases the odds of high burden by 2-fold, and cessation of actions increases the odds of caregiver burden by 3.5-fold.

In Model II, a poorer quality of life in the social domain odds ratio (OR) = 0.773; 95% confidence interval (CI) 0.621–0.950; (<0.0169); and environmental domain odds ratio (OR) = 0.680; 95% confidence interval (CI) 0.470–0.955; (<0.0316); determine a high caregiver burden, and in the psychological domain there is an almost 2-fold increase in the chance of a high burden odds ratio (OR) = 1.774; 95% confidence interval (CI) 1.238–2.684; (<0.0034). The Gini coefficient indicates in Model I that it is more important to stop actions than to discharge them. On the other hand, given both models, functioning in the environment is the most important characteristic that contributes to the level of caregiver burden. Patient age is the only one of the sociodemographic factors studied that significantly affects the occurrence of high levels of caregiver burden odds ratio (OR) = 1.894; 95% confidence interval (CI) 1.200–3.217; (<0.0105). It increases the chance of high levels of caregiver burden by two times. A higher degree of disability rating increases the chance of a high level of caregiver burden by three times; odds ratio (OR) = 2.726; 95% confidence interval (CI) 1.462–6.736 (<0.0075).

The occurrence of mental exhaustion, feelings of frustration, time spent caring for a loved one, and financial situation all have an impact on a high caregiver burden. Mental exhaustion: odds ratio (OR) = 20.181; 95% confidence interval (CI) 3.534–174.109; (<0.0018); frustration odds ratio (OR) = 10.230; 95% confidence interval (CI) 1.587–94.655; (<0.0212); time spent caring: odds ratio (OR) = 1.645; 95% confidence interval (CI) 1.082–2.636; (<0.0264); material situation: odds ratio (OR) = 0.247; 95% confidence interval (CI) 0.119–0.437; (<0.0001). Detailed data are shown in Table 4.

The analysis conducted, using a logistic regression analysis, showed that the caregiver burden is influenced by parameters such as quality of life, stress, caregiver-triggered strategies (discharge and cessation of activities), caregiver frustration, psychological burden, financial situation, longer time spent on patient care, patient functional status (ADL), significant degree judgment of disability, and age of the patient. This is presented in Table 5.

**Figure 2:**
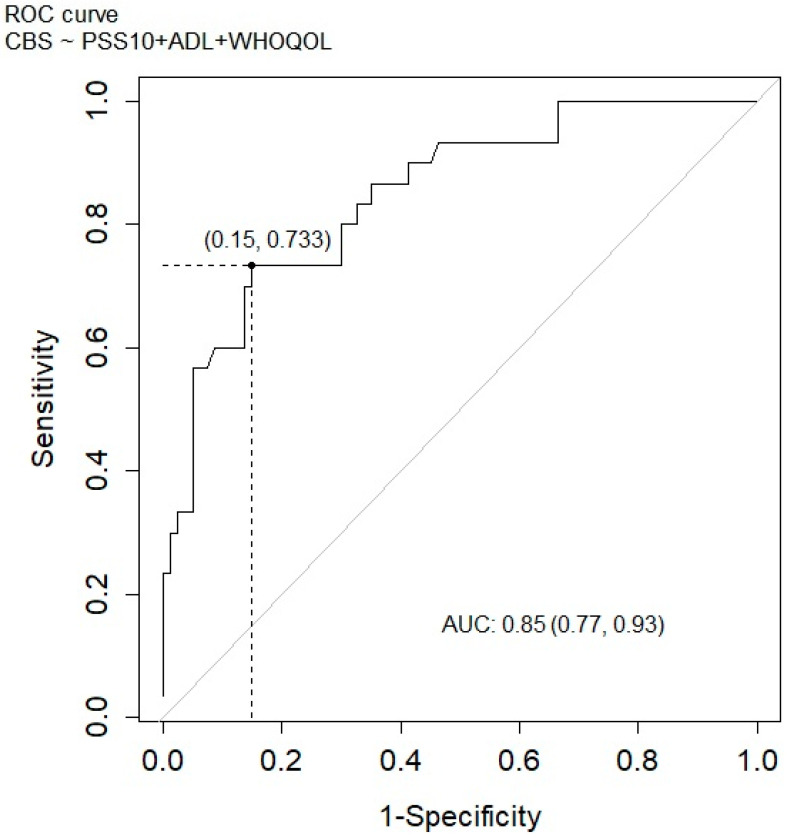
ROC curve for high-burden caregivers including stress level, ADL scale, and quality of life.

For a cut-off value of 0.5, the area under the ROC curve (AUC) is much larger than 0.5 and is 0.85. Based on the analysis, it can be concluded that the model is a very good fit to the data. The 95% CI (confidence interval) for the accuracy is (0.77, 0.93). All this can be graphically seen in Figure 2.

Sensitivity, for the model with a cut-off point of 0.273, is 73.3%, while the specificity is 70%. It can be concluded that the calculated model will better predict respondents recognized by the model as high-burden caregivers than caregivers classified as low- or medium-burden. A model with a cut-off point of 0.5 will be a model with a better fit to the data; however, this may contribute to the misclassification of a caregiver into a high-burden level.

## 4. Discussion

An important determinant of the aftermath of a stroke is the subjective assessment of quality of life. Among the various factors that can affect the quality of life of caregivers, one of the important ones associated with stroke seems to be the burden of caring for the patient. The burden in turn may depend on factors on the part of the caregiver as well as the patient. On the basis of the results of our study, we can conclude that the determinants affecting a high level of caregiver burden are as follows: low quality of life of the caregiver resulting from the burden, stress, strategies activated by the caregiver (discharge and cessation of activities), frustration of the caregiver, psychological burden, material situation, longer time spent on caring for the patient, functional status (ADL) on the part of the patient, a significant degree of disability, and the age of the patient. It should be added that these are the few scientific reports that indicate determinants of a high burden of caregiving for post-stroke patients in the first six months after a stroke event [14,26]. In our study, the caregivers’ QoL was altered in all domains of the questionnaire. There was a lower rate of satisfaction with the aspects that make up the physical domain (12.1 ± 1.7). Similar results were obtained by Silva et al. [27], indicating the lowest value of the physical domain. Similar results were found in a study carried out with caregivers of post-stroke individuals; the physical domains were the most affected [28]. The analysis of correlations between caregiver burden and the QoL domains of the WHOQOL-BREF show correlations with all domains: social, physical, and psychological, environment, all domains. The study by Silva et. al. similarly correlates with caregiver burden in our study with the exception of the environment domain. In the presented own research by means of logistic regression, the quality of life of caregivers is a significant characteristic (Gini mean coefficient 12.979) influencing a high caregiver burden, in particular in the environment domain.

In a logistic regression analysis of our own research, we found that stress, lower quality of life, and lower functional capacity determine a high burden on the caregiver of a stroke survivor. Lee et.al [14] presented a similar study considering high burdens. The number of caregivers who were assigned to high levels of burden was similar to their study (37.9% vs. 27.3%), and the determinants of a high burden were similar. A multivariable regression analysis with significant factors was performed to evaluate caregiver burden predictors at 6 months. The following were identified as independent caregiver burden predictors at 6 months: patients’ disability (*p* = 0.016), caregivers’ self-rated stress (*p* = 0.013), and caregivers’ quality of life (EQ-5D) (*p* = 0.042). Due to the abrupt onset of disability and the chronic nature of stroke recovery, caring for a stroke survivor has been found to have a negative impact on the physical, mental, and psychological health of caregivers [29,30]. Primary caregivers of stroke patients tend to report more somatic and depressive symptoms, sleep disorders, stress, and social isolation than the general population [31].

In our study, caregivers who were high-burden were accompanied by frustration, fatigue, and allocated a longer time during the day to caregiving. Patients were significantly limited in motor skills. It should also be added that in assessing the quality of life of caregivers, the psychological domain increases the chance of high burden by two times. A lower quality of life in terms of social and environmental domains determines a high caregiver burden. In a similar study by Kaseke et.al [32], caregivers reporting feeling completely overwhelmed had a significantly higher CBS at 3 months (*p* < 0.001) compared to those not completely overwhelmed. In addition, caregivers providing care to stroke survivors with poor community reintegration reported high median CBS scores of 9 (IQR: 5, 11) at 3 months Caregivers attending to stroke survivors with very severe motor impairments had the highest CBS median score of 11 (IQR: 9, 12) at 3 months.

In our study, it was proven that the accompanying stress of caregivers of post-stroke patients is a determinant of a high caregiver burden. Similar findings were presented by Sohkhlet et al. [33] among caregivers of post-stroke families, where the average duration of care was 6 months; the stress they determined was related to the self-sufficiency of the patient. Assistance with activities of daily living increased caregivers’ stress levels. The accompanying stress increased the caregiver’s burden, worsening their quality of life. Activating strategies that may act destructively or constructively in coping with the accompanying stress may be a way for the caregiver to cope better or worse in stressful situations. The authors of this study set out to see how, and if at all, individual domains of the Mini-Cope questionnaire were affected. Logistic regression analysis proved that of the 14 domains, 2 of them (discharge and cessation) negatively influence caregivers, increasing the chances of a high caregiver burden. In a similar study by Kazemi et al. [34], caregivers with a higher burden of care used more negative coping strategies, such as escape–avoidance and distancing. In order to encourage caregivers to utilize effective coping skills, appropriate programs should be designed and implemented to support caregivers. The use of effective coping skills to reduce the level of personal burden can improve caregivers’ physical health and psychological well-being. Informal caregivers with a higher burden of care used emotion-focused strategies, which often do not help in reducing caregiver stress. As such, training programs that teach caregivers efficient coping strategies are needed in order to increase their use of effective and healthy coping strategies.

Another determinant affecting a high caregiver burden was the assessment of daily living activities (ADL). Logistic regression proved that if daily living activities were fully preserved, the chance of a high caregiver burden was 35.4% lower. Rawat et al. [35] reported that more than half (56.67%) of stroke caregivers felt exhausted (high/extreme burden). The reason for these different results in the care burden of caregivers of stroke patients may be related to the degree of dependence of the patients for their daily living activities. In their study, Baumann et al. [36] found that a decrease in patients’ ability to perform daily activities was significantly associated with an increase in the care burden of caregivers of stroke patients. One might postulate that caregivers living together with the patient spend more time with actually assisting the patients with daily tasks. Indeed, in the literature, it is reported in an Australian study including 71 caregivers that at 6 months post stroke, 61% of caregivers of stroke patients spend on average 4.6 h helping the patient with basic and instrumental activities of daily living and the household per day [37].

The only sociodemographic factor of the post-stroke patient that affected the high caregiver burden was the patient’s age. which significantly affects the occurrence of the high caregiver burden odds ratio (OR) = 1.894; 95% confidence interval (CI) 1.200–3.217; (<0.0105), It increases the chance of a high caregiver burden by two times. It is an ambiguous factor in the literature. Some authors have shown no effect of this factor on quality of life and caregiver burden [38], while others have shown that it worsens as the patient’s age increases [39]. In contrast, Grant et al. showed that the older the patient, the faster over time caregivers’ quality of life deteriorates [40]. Due to the few scientific reports indicating determinants of a high caregiver burden, they seem to be justified; larger sample size and multivariate analyses are needed for a better investigation of the factors associated with a high caregiver burden.

The study presented here has provided information about the extent of the problem of high levels of caregiver burden and which factors determine it. The results may indicate the need to expand our knowledge of stroke care, develop a management plan, pay attention to the rights of post-stroke patients, and establish a common scope for the interdisciplinary activities of medical and social care personnel supporting caregivers of post-stroke patients, as well as to determine the type of necessary support for informal caregivers, including the organization of caregivers and patients, and popularize day care centers.

### 4.1. Limitation

This study was conducted at a single time point, so changes over time could not be analyzed and improvement in the patients and their caregivers could not be assessed in such a protocol. Given that the first months of providing care for a patient are the most difficult, the results obtained seem to constitute a valuable scientific study. This study did not have a control group to evaluate the impact of the care provided on the caregivers’ quality of life. This limitation was minimized by comparing the data obtained to scientific reports available in the literature. Methodological limitations, on the one hand, limit the interpretation of the obtained results, which, on the other hand, may provide important clues for further research.

### 4.2. Benefits

In Poland, this is the first study and one of few in Europe and the world to simultaneously assess the burden, stress levels, depression, activated coping methods, and quality of life of caregivers, considering the high level of caregiver burden at 6 months after ischemic stroke. First, this study shows the need to pay attention to the level of caregiver burden, including the incidence of depression in caregivers. It also brings to light the need to assess caregivers’ need for different types of support. Finally, it signals the legitimacy of introducing systemic solutions through the introduction of systematic educational programs within stroke units, the introduction of social campaigns, and the introduction of day rehabilitation units. This study draws attention to the fact that the consequences of stroke are serious not only for patients but also for their caregivers.

## 5. Conclusions

A level of high burden was reached by 27.3% of caregivers caring for a sick family member after a stroke. This study highlights the significant influence of factors from both the caregiver and the patient. Quality of life is one of the important characteristics of the caregiver that influences the high level of caregiver burden. A worse quality of life in the social and environmental domains determines a high caregiver burden, and in the psychological domain, the chance of occurrence of a high burden increases by almost two times. Increased stress levels, activation of the discharge and cessation domain, longer time devoted to the patient’s material situation, and frustration and exhaustion of the caregiver significantly affect the occurrence of a high caregiver burden. The patient’s age, significant disability rating, and independence affect the high burden on the caregiver. Seeking systemic solutions through psychologist support, social campaigns, the introduction of day rehabilitation units, and perhaps “stroke navigators” to help adapt to the caregiver’s role and alleviate the associated burden, taking into account both the caregiver’s and the patient’s needs, will improve the relationship and help in their daily functioning.

## Figures and Tables

**Table 1:** Characteristics of caregivers of stroke patients and patients among groups of caregivers with high caregiver burden.

Characteristics		Caregivers		Patients	
		Total (*n* = 110)	Including Caregivers with High Levels of Burden (*n* = 30)	Total (*n* = 110)	Including Patients Who Are a High Burden on Their Caregivers (*n* = 30)
Gender	Female	83 (75.5%)	22 (73.3%)	63 (57.3%)	15 (50.0%)
	Male	27 (24.5%)	8 (26.7%)	47 (42.7%)	15 (50.0%)
Age	<50	32 (29.2%)	8 (26.7%)	5 (4.5%)	1 (3.3%)
	51–60	36 (32.7%)	9 (30.0%)	6 (5.5%)	0 (0.0%)
	61–70	25 (22.7%)	6 (20.0%)	25 (22.7%)	4 (13.3%)
	71–80	15 (13.6%)	7 (23.3%)	35 (31.8%)	8 (26.7%)
	>80	2 (1.8%)	0 (0.0)	39 (35.5%)	17 (56.7%)
Education	Primary	2 (1.8%)	1 (0.4%)	17 (15.5%)	6 (20.0%)
	Vocational	38 (34.5%)	10 (33.3%)	49 (44.5%)	10 (33.3%)
	Secondary	30 (27.3%)	10 (33.3%)	30 (27.3%)	10 (33.3%)
	Higher	40 (36.4%)	9 (30.0)	14 (12.7%)	4 (13.3%)
Marital status	Single	23 (20.9%)	6 (20.0%)	52 (47.3%)	12 (40.0%)
	In a relationship	87 (78.2%)	24 (80.0%)	58 (52.7%)	18 (60.0%)
Place of residence	Rural	47 (42.7)	13 (43.3)	46 (41.8%)	12 (40.0%)
	City	63 (57.3%)	17 (56.7%)	64 (58.2%)	18 (60.0%)
Source of income	Professional work	68 (61.8%)	17 (56.7%)	0 (0.0%)	3 (10.0%)
	Pension	37 (33.7%)	10 (30.3%)	102 (92.8%)	26 (86.7%)
	Other (allowance; no income)	5 (4.5%)	3 (10.0%)	8 (7.2%)	1 (3.3%)
Financial situation (mean ± SD)		3.48 ± 0.62	3.33 ± 0.66	2.62 ± 0.66	2.66 ± 0.66
Duration of care (for patient)	0.5 years	17 (15.4%)	2 (6.7%)	17 (15.5%)	3 (10.0%)
	0.5–1 years	20 (18.2%)	3 (10.0%)	22 (20.0%)	5 (16.7%)
	1–2 years	21 (19.1%)	9 (30.0%)	15 (13.6%)	5 (16.7%)
	2–5 years	22 (20.0%)	6 (20.0%)	23 (20.9%)	5 (16.7%)
	>5 years	30 (27.3%)	10 (33.3%)	33 (30.0%)	12 (40.0%)
Average time spent per day on care	1–2 h	26 (23.6%)	2 (6.6%)	Degree of disability (n = 99)	
	3–5 h	27 (24.5%)	8 (26.7)	mild	
	5–10 h	28 (25.5%)	8 (26.7)	14 (12.7%)	0 (0.0%)
	>10 h	29 (26.4%)	12 (40.0)	moderate	
Relationship	husband or wife	38 (34.6%)	9 (30.0%)	12 (10.9%)	2 (6.9%)
	mother or father	53 (48.2%)	16 (53.3%)	severe	
	mother-in-law father-in-law	5 (4.5%) 14 (12.7%)	2 (6.7%) 3 (10.0%)	73 (66.4%)	27 (93.1%)
Change in financial situation (mean value ± standard deviation)		2.38 ± 0.62	2.07 ± 0.58		
Other indicators mean ± SD (range of scores: minimum value–maximum value)					
Caregiver Burden Scale		2.39 ± 0.74 (1.00–3.73)	3.26 ± 0.20 (3.00–3.72)		
Beck Depression Inventory		12.32 ± 9.29 (0–44)	18.77 ± 10.02 (0–44)		
Perceived Stress Scale		20.91 ± 4.62 (3–29)	23.40 ± 3.20 (14–28)		
Total score (by WHOQOL-BREF)		13.39 ± 1.97 (8.67–18.17)	12.04 ± 2.03 (8.67–16.50)		
The modified Rankin Scale				3.76 ± 1.35 (0.00–5.00)	4.47 ± 0.90 (2.00–5.00)
Activities of Daily Living Scale				2.59 ± 2.20 (0.00–6.00)	1.20 ± 1.47 (0.00–5.00)

Notes: Material situation: very good (5), good (4), average (3), bad (2), very bad (1). Change in material situation: very deteriorated (1), partially deteriorated (2), no change (3), somewhat improved (4), definitely improved (5).

**Table 2:** Caregiver burden according to CBS scale.

Caregiver Burden Scale	M	SD	Me	Q1–Q3	Min	Max	Level of Burden		
							Low	Medium	High
General burden	2.64	0.82	2.81	2.00–3.38	1.00	4.00	26 (23.6%)	34 (30.9%)	50 (45.5%)
Social isolation	2.42	0.96	2.67	1.67–3.33	1.00	4.00	39 (35.5%)	28 (25.5%)	43 (39.1%)
Disillusionment	2.41	0.81	2.60	1.80–3.00	1.00	4.00	36 (32.7%)	43 (39.1%)	31 (28.2%)
Emotional involvement	2.03	0.85	2.00	1.33–2.67	1.00	4.00	49 (44.5%)	38 (34.5%)	23 (20.9%)
Surrounding	2.03	0.71	2.00	1.33–2.67	1.00	3.67	48 (43.6%)	45 (40.9%)	17 (15.5%)
Total	2.39	0.74	2.45	1.73–3.00	1.00	3.73	36 (32.7%)	44 (40.0%)	30 (27.3%)

Notes: M—mean, SD—standard deviation, Q1—quartile 25%, Me—median, Q1—quartile 75%, Min—minimum value, Max—maximum value.

**Table 3:** Determinants of caregiver burden.

		Caregiver Burden Scale				
		General Burden	Social Isolation	Disappointment	Emotional Involvement	Environment
Perceived Stress Scale ver. 10		0.546 ***	0.501 ***	0.461 ***	0.397 ***	0.313 ***
MINI-COPE	Active coping	0.117	0.120	0.032	0.111	0.068
	Planning	0.089	0.063	0.039	0.000	0.044
	Positive re-evaluation	0.074	−0.002	0.012	0.106	−0.113
	Acceptance	0.016	−0.033	−0.042	0.045	−0.023
	Sense of humor	0.061	0.113	0.097	0.136	0.067
	Turning to religion	0.044	−0.098	−0.050	0.033	−0.028
	Seeking emotional support	−0.199 *	−0.173 ^ǂ^	−0.133	−0.156	−0.091
	Seeking instrumental support	−0.041	−0.087	−0.062	−0.118	−0.052
	Preoccupation with something else	0.235 *	0.278 **	0.219 *	0.281 **	0.211 *
	Denial	0.401 ***	0.428 ***	0.512 ***	0.348 ***	0.368 ***
	Discharging	0.363 ***	0.343 ***	0.292 **	0.237 *	0.180 ^ǂ^
	Using psychoactive substances	0.187 ^ǂ^	0.181 ^ǂ^	0.214 *	0.132	0.070
	Discontinuing activities	0.412 ***	0.339 ***	0.470 ***	0.242 *	0.346 ***
	Blaming yourself	0.400 ***	0.342 ***	0.418 ***	0.197 *	0.403 ***
Beck Depression Scale		0.561 ***	0.472 ***	0.500 ***	0.327 ***	0.406 ***
WHOQOL-BREF	What is your quality of life? ^1^	−0.390 ***	−0.340 ***	−0.393 ***	−0.175 **	−0.299 ***
	Are you satisfied with your life? ^1^	−0.400 ***	−0.402 ***	−0.440 ***	−0.177 **	−0.317 ***
	Somatic Domain	−0.368 ***	−0.269 **	−0.331 ***	−0.270 **	−0.357 ***
	Psychological Domain	−0.454 ***	−0.410 ***	−0.401 ***	−0.190 *	−0.385 ***
	Social Domain	−0.540 ***	−0.473 ***	−0.536 ***	−0.362 ***	−0.478 ***
	Environmental Domain	−0.486 ***	−0.464 ***	−0.522 ***	−0.286 **	−0.414 ***
	Total	−0.532 ***	−0.483 ***	−0.542 ***	−0.314 **	−0.475 ***
The modified Rankin Scale		0.382 ***	0.484 ***	0.423 ***	0.235 *	0.255 **
Activities of Daily Living Scale		−0.424 ***	−0.464 ***	−0.450 ***	−0.282 **	−0.280 **
Abbreviated Mental Test Score		−0.460 ***	−0.530 ***	−0.492 ***	−0.447 ***	−0.316 *

Notes: *** < 0.001, ** < 0.01, * < 0.05, ^ǂ^ 0.1; ^1^ Kendall’s tau correlation coefficient; otherwise Spearman’s rank correlation coefficient.

**Table 4:** Logistic regression analysis of high caregiver burden in stroke patients measured for a period of six months after stroke event.

	Coeff.	Standard Error	Wald’s Test	*p*-Value	Odds Ratio	95% CI for Odds Ratio		Gini Index
						2.5%	97.5%	
General model based on standardized questionnaires—patients/caregivers								
PSS10	0.181	0.050	3.641	<0.0001	1.198	1.096	1.334	10.864
ADL	−0.437	0.159	−2.738	0.006	0.646	0.458	0.863	8.632
WHOQOL	−0.074	0.020	−3.673	<0.0001	0.928	0.889	0.963	16.103
AIC = 97.219 LR test: χ^2^ = 61.273 df = 3 *p* < 0.0001 HL test: χ^2^ = 11.81 df = 8 *p* = 0.160 R^2^_McFadden_ = 0.292								
Caregivers								
Model I based on the domains of the MINI-COPE questionnaire								
(Intercept)	−3.351	0.724	−4.629	<0.0001				
Venting	0.848	0.379	2.235	0.0254	2.335	1.134	5.115	6.909
Resignation	1.308	0.407	3.213	0.0013	3.699	1.750	8.785	10.713
AIC = 113.68 LR test: χ^2^ = 12.552 df = 1 *p* = 0.0004 HL test: χ^2^ = 11.471 df = 8 *p* = 0.1764 R^2^_McFadden_ = 0.165								
Model II based on the domains of the WHOQOL-BREF questionnaire								
Psychological	0.574	0.196	2.929	0.0034	1.774	1.	2.684	9.979
Social	−0.257	0.107	−2.389	0.0169	0.773	0.621	0.950	10.593
Environmental	−0.386	0.179	−2.149	0.0316	0.680	0.470	0.955	12.979
AIC = 115.24 LR test: χ^2^ = 43.254 df = 3 *p* < 0.0001 HL test: χ^2^ = 7.119 df = 8 *p* = 0.524 R^2^_McFadden_ = 0.152								
Model based on self-reported survey								
SYT_MAT	−1.398	0.328	−4.263	<0.0001	0.247	0.119	0.437	4.629
TIME	0.498	0.224	2.221	0.0264	1.645	1.082	2.636	4.639
DIS_NEU	2.106	0.781	2.695	0.0070	8.215	1.849	42.030	4.375
FEEL_ANG	2.325	1.009	2.304	0.0212	10.230	1.587	94.655	3.524
FEEL_PSYCH	3.005	0.962	3.125	0.0018	20.181	3.534	174.109	5.227
AIC = 94.702 LR test: χ^2^ = 67.791 df = 5 *p* < 0.0001 HL test: χ^2^ = 8.621 df = 7 *p* = 0.2811 R^2^_McFadden_ = 0.343								
Patients								
Model based on sociodemographic data								
(Intercept)	−3.571	1.075	−3.321	0.0009				
AGE	0.639	0.250	2.558	0.0105	1.894	1.200	3.217	4.843
AIC = 124.96 LR test: χ^2^ = 7.945 df = 1 *p* = 0.0048 HL test: χ^2^ = 3.314 df = 3 *p* = 0.3457 R^2^_McFadden_ = 0.062								
Model based on degree of disability								
(Intercept)	−3.565	1.066	−3.344	0.0008				
DISAB	1.003	0.376	2.671	0.0075	2.726	1.462	6.736	4.914
AIC = 120.85 LR test: χ^2^ = 12.061 df = 1 *p* = 0.0005 HR test: χ^2^ = 2.745 df = 2 *p* = 0.2535 R^2^_McFadden_ = 0.094								

Notes: PSS10—Perceived Stress Scale, ADL—Activities of Daily Living Scale, WHOQOL—total quality of life, SYT_MAT—change in caregiver’s financial situation, TIME—time spent caring for a loved one, DIS_NEU—caregiver suffering from neurosis, FEEL_ANG—caregiver’s perceived frustration and anger, FEEL_PSYCH—caregiver’s mental exhaustion, AGE—patient’s age, DISAB—degree of disability rating, LR—likelihood ratio test, HL—Homser–Lemeshowa test, *p*—level of significance.

**Table 5:** The number of correct and incorrect predictions for two cut-offs: 0.273 and 0.5.

	Confusion Matrix					
	A Cut-Off Value of 0.273			A Cut-Off Value of 0.5		
Caregiver Burden Scale	Predicted		Total	Predicted		Total
	High Level of Caregiver Burden	Low/Medium Level of Caregiver Burden		High Level of Caregiver Burden	Low/Medium Level of Caregiver Burden	
Observed						
High-level caregiver burden	22 (TP)	8 (FN)	30	17 (TP)	13 (FN)	30
Low/medium-level caregiver burden	24 (FP)	56 (TN)	80	4 (FP)	76 (TN)	80
Total	46	64	110	21	89	110

Notes: TP: true positive, FP: false positive, TN: true negative, FN: false negative.

## Data Availability

The raw data supporting the conclusions of this article will be made available by the authors on request.

## Abbreviations

The following abbreviations are used in this manuscript:

ADL	Activities of Daily Living
AMTS	Abbreviated Mental Test Score
AUC	Area under Receiver Operating Characteristic Curve
BDI	Beck Depression Inventory
CBS	Caregiver Burden Scale
GBD	Global Burden of Diseases, Injuries, and Risk Factors Study
mRS	The modified Rankin Scale
PPS-10	Perceived Stress Scale
QoL	Quality of life
ROC	Receiver Operating Characteristic Curve
SD	Standard deviation
WHOQoL-BREF	WHO Quality of Life Brief Version
